# Plasma Lipids and Lipoproteins in Sickle Cell Disease Patients in the Northern West Bank, Palestine

**DOI:** 10.1155/2021/6640956

**Published:** 2021-08-04

**Authors:** Fekri Samarah, Mahmoud A. Srour, Kamal Dumaidi

**Affiliations:** ^1^Department of Medical Laboratory Sciences, Faculty of Allied Health Sciences, Arab American University, State of Palestine; ^2^Department of Biology and Biochemistry, Faculty of Science, Birzeit University, Birzeit, State of Palestine

## Abstract

**Background:**

Lipid metabolism may be altered in red cell genetic disorders. The erythrocyte and plasma lipids are defected which may increase the risk of cardiovascular disease. In the present study, we hypothesized a possible link between severity of anemia and altered lipid profile in SCD.

**Methods:**

A total of 151 SCD patients, including 62 patients with sickle cell anemia (SS), 54 patients with sickle *β*-thalassemia (ST), and 35 individuals with sickle cell trait (AS), were studied. The control group consisted of 160 healthy individuals. Total cholesterol (TC), triglyceride (TG), and high-density lipoprotein cholesterol (HDL-C) were enzymatically measured.

**Results:**

Total cholesterol and LDL-C were significantly lower (*P* value < 0.001) in SS and ST patients compared to AS individuals and AA controls. However, LDL-C was significantly lower in AS individuals (both males and female) compared to AA controls (*P* value < 0.001). The HDL-C in SS and ST patients (both males and females) was significantly lower than that in AS individuals (*P* value < 0.001). In addition, the HDL-C was significantly higher in SS and ST males and AS (males and females) compared to AA controls (*P* value < 0.001). The HDL-C was also significantly higher in SS males (*P* value < 0.001) and females (*P* value < 0.05) compared to ST patients. The HDL-C was significantly higher in AS individuals (*P* value < 0.001) compared to AA controls. The triglycerides in SS males was significantly lower than that in ST patients (*P* value < 0.001), but there was no significant difference when compared to AS individuals and AA controls. In contrast, triglycerides in SS females were significantly lower than those in ST (*P* value < 0.05), AS (*P* value < 0.001), and AA controls (*P* value < 0.001). In males of ST patients, triglycerides were significantly higher than those observed in AS males and AA males (*P* value < 0.001). In contrast, females of ST patients have a significantly lower triglycerides compared to AS and AA females (*P* value < 0.001).

**Conclusions:**

In SCD, the plasma is affected in some way, especially the plasma cholesterol that was investigated in this study. Further prospective studies should examine the contribution of an altered lipid profile to the severity and clinical complications in SCD patients.

## 1. Introduction

Beta-thalassemia and sickle cell disease (SCD) are among the most common inherited abnormalities of hemoglobin synthesis in Palestine. The prevalence of *β*-thalassemia trait in the West Bank and in Gaza Strip of Palestine is 3.1% and 4.3%, respectively [1]. The prevalence of a sickle cell trait in the West Bank region of Palestine is estimated to be 1.2% [2]. In Palestine, homozygotes for HbS (*β*6(A3) Glu→Val, GAG>GTG) are accompanied by low levels of HbF and sever clinical course that generally leads to a shortened life span [3]. Sickle cell disease (SCD) denotes all genotypes containing at least one sickle gene. The disease can be inherited in combination with another hemoglobinopathy such as *β*-thalassemia (*β*^0^ or *β*^+^), with varying degrees of severity depending on the amount of HbA production [4]. Sickle/*β*^0^-thalassemia (S/*β*^0^) resembles sickle cell anemia (Hb SS) and can be differentiated by elevated HbA2 concentrations, low MCV, and familial studies [5]. From a clinical viewpoint, SCD manifestations can be roughly attributed to two phenomena: hemolysis and vasoocclusion, disturbing microcirculation resulting in oxidative and inflammatory stress [6]. The vasculopathy seen in SCD is similar to that of atherosclerosis and coronary heart disease [7, 8] where lipid monitoring is an important guide. It is reported that in SCD besides elevated markers of endothelial dysfunction, such as vascular cell adhesion molecule-I, interleukin-6, C-reactive protein, selectins and vascular endothelial growth factor, decreased apolipoprotein A-1 (apoA-1) levels, reduced nitric oxide (NO) bioavailability, and decreased HDL-C are similar in both diseases. The vasculopathy observed in SCD is not attributable precisely to total cholesterol (TC) and low-density lipoprotein cholesterol (LDL-C) levels, since both have been shown to be decreased in SCD patients compared to healthy individuals. Even though LDL-C levels are diminished in SCD, the proportion of small dense LDL is an important factor to consider in SCD vasculopathy, since it can easily leak into the subendothelial space, be held by proteoglycans, and easily be oxidized [9].

There is epidemiologic evidence that hypocholesterolemia (<135 mg/dL) is associated with an increase in all-cause mortality rates [10]. In addition, hypocholesterolemia (<147 mg/dL) has been associated with increased risk of all-cause mortality among patients with coronary heart disease [11] or stroke, heart disease, and cancer [12]. In another study, release of iron and radical formation resulting in membrane lipid bilayer destruction and lipid peroxidation and elevated markers of endothelial dysfunction have been reported [13, 14]. Previous studies showed a lower plasma cholesterol levels in SCD patients compared to healthy controls [9, 12, 13, 15–17]. The literature contains few reports that address cholesterol levels in SCD patients. So, the aim of the present study is to determine the serum lipid profiles in Palestinian SCD patients, to compare the results with sex- and age-matched healthy controls and sickle cell trait (AS) subjects, and to find any relationship between plasma lipid profiles and hematological indices.

## 2. Materials and Methods

### 2.1. Participants and Study Design

In this cross-sectional case control study, one hundred and fifty-one sickle cell disease (SCD) patients, attending the Thalassemia Governmental Units (Nablus, Tulkarm, and Jenin) in the northern West Bank, Palestine, were selected to participate in this study. Furthermore, one hundred sixty age- and sex-matched healthy individuals were randomly selected from the same region. Participants with SCD diagnosed by Hb electrophoresis and molecular studies were assessed at a steady state with no vasooclussive crisis, had not experienced an episode of acute chest syndrome, without acute complications related to the disease or infectious conditions, and had no blood transfusion in the last 4 weeks. Healthy individuals were of similar age, were not taking any medications, and had a normal Hb electrophoresis pattern. Only healthy control individuals who were not obese (body mass index (BMI) < 25) were selected. Individuals with SCD were excluded if they had a stroke or blood transfusion 4 weeks before the study. Healthy subjects were excluded if they have chronic disease, acute infectious, dyslipidemia, and obesity. Seventy-one of 116 (61.2%) SCD patients (SS and ST) were undergoing hydroxyurea (HU) therapy. None of AS subjects was under HU treatment. Ethical approval for the study was obtained from the Ministry of Health (MOH) in Palestine, and all participants gave oral informed consent. The sickle cell phenotype in sickle cell anemia (SS) patients was assessed by Hb electrophoresis and confirmed by RFLP PCR. The phenotype of sickle *β*-thalassemia patients (ST) was assessed by Hb electrophoresis and confirmed by DNA analysis of the *β*-globin gene. The genotype of the sickle cell trait patients was determined by RFLP PCR. Concerning the control subjects, the absence of a sickle cell trait and *β*-thalassemia trait was confirmed by the presence of a normal Hb electrophoresis profile.

### 2.2. Anthropometry

Anthropometric parameters (weight, height, and body mass index (BMI)) were evaluated in all recruited subjects. Weight and height were measured using a weighing scale and stadiometer, respectively. BMI was calculated as (weight in kg/height in meters squared). Age- and sex-specific BMI percentiles of participants were categorized as underweight: <5^th^ percentile, normal weight: >5^th^, <85^th^ percentile; overweight: ≥85^th^ percentile; and obese: >95^th^ percentile [18].

### 2.3. Laboratory Measurements

Fasting blood samples were obtained from all participants for determination of experimental parameters. Venous blood was collected in EDTA tubes and used for determination of hematological indices including red blood cell count (RBC), hemoglobin concentration, and mean cell volume (MCV) using Nihon Kohden (ME6510K) (Diamond Diagnostics, Japan) cell counter. Another blood sample was collected using a plain tube (red cap) and used for determination of plasma lipids. The tube was centrifuged for 10 minutes at 3000 rpm. Then, the serum sample was separated into a sterile Eppendorf tubes and stored at -20 ^O^C until being analyzed. Diagnosis of SCD in patients was based on clinical, familial, and laboratory estimation of HbF and HbA2 levels and *β*-globin gene mutation analysis. Sickle cell phenotypes were diagnosed by conventional electrophoresis methods (cellulose acetate at alkaline and acid pH) [19]. Homozygosity for the *β*^S^-mutations was ascertained by polymerase chain reaction (PCR) followed by digestion with the restriction enzyme DdeI [20].

Total cholesterol (TC), triglyceride (TG), and high-density lipoprotein cholesterol (HDL-C) were measured enzymatically using ChemWell chemistry analyzer (Awareness Tech, USA). Low-density lipoprotein cholesterol (LDL-C) levels were estimated from the Friedewald formula (FF): (LDL‐C = TC–[HDL‐C + (TG/5)]).

## 3. Statistical Analysis

Descriptive statistics including mean and standard deviation (SD) were calculated for all variables using SPSS software (version 23). Inferential statistics were used to reach conclusions and generalize about the characteristics of populations based on laboratory measurements of study samples. These statistical tests include one-way ANOVA and post hoc LSD. A *P* value < 0.05 was considered statistically significant.

## 4. Results

One hundred and fifty-one participants with SCD and Hb AS trait and 160 healthy controls were recruited from the Thalassemia Governmental Units (Nablus, Tulkarm, and Jenin) in the northern West Bank, Palestine. They were distributed as follows: 62 homozygous Hb SS patients (30 males with a mean age 18.7 years); 32 females (with a mean age of 21.2 years); 54 sickle/*β*-thalassemia (39 sickle/*β*^0^-thalassemia and 15 sickle/*β*^+^-thalassemia) including 21 females (mean 19.2 years) and 33 males (mean 16.4 years); and 35 sickle cell trait individuals, including 21 males (mean 22.4 years) and 14 females (mean 20.9 years). Furthermore, one hundred sixty age- and sex-matched healthy individuals, including 89 males (mean 21.5 years) and 71 females (mean 21.9 years) were randomly selected from the same region.

There was no statistically significant difference between the average age of SCD patients and controls (Table 1). Overall, a higher proportion of participants are adolescents (≥14 years) (late teens to early 20s). There were more males compared to females (55.6 vs. 44.4). The proportion distribution of sickle Hb phenotype between males and females did not show significant differences (*P* value = 0.157). SCD patients with sickle cell anemia (SS) had significantly reduced BMI compared with Hb AS (*P* value < 0.05). A higher proportion of patients with Hb SS phenotype (14.5%) was underweight compared to the ST phenotype (5.6%). BMI status was significantly associated with Hb phenotype (*P* value < 0.001) (Table 1).

The hematological parameters (Hb, RBC count, and MCV; Table 2) and lipid profile (total cholesterol, LDL-C, HDL-C, and triglycerides; Table 3) of each group of SCD patients (SS, ST, and SA) were compared to each other and to the controls (AA). Mean comparisons were performed separately between the gender groups in patients and controls.

The Hb and RBC count were significantly lower in SS and ST patients (both males and females) than those in SA individuals (*P* value < 0.001) and AA controls (*P* value < 0.001). The MCV of SS patients (both males and females) were higher than that observed in ST, AS, and AA controls (*P* value < 0.001). However, comparison of AS individuals with AA controls showed that only AS males have a significantly lower Hb (*P* value < 0.001) and RBC count (*P* value < 0.05) compared to AA controls.

Total cholesterol and LDL-C were significantly lower (*P* value < 0.001) in SS and ST patients compared to AS individuals and AA controls (Table 3).

The LDL-C was significantly lower in AS individuals (both males and female) compared to AA controls (*P* value < 0.001). The HDL-C in SS and ST patients (both males and females) was significantly lower than that in AS individuals (*P* value < 0.001). In addition, the HDL-C was significantly higher in SS and ST males and AS (males and females) compared to AA controls (*P* value < 0.001). The HDL-C was also significantly higher in SS males (*P* value < 0.001) and females (*P* value < 0.05) compared to ST patients. The HDL-C was significantly higher in AS individuals (*P* value < 0.001) compared to AA controls.

The triglycerides in SS males were significantly lower than that in ST patients (*P* value < 0.001), but there was no significant difference when compared to AS individuals and AA controls. In contrast, triglycerides in SS females were significantly lower than that in ST (*P* value < 0.05), AS (*P* value < 0.001), and AA controls (*P* value < 0.001). In males of ST patients, triglycerides were significantly higher than that observed in AS males and AA males (*P* value < 0.001). In contrast, females of ST patients have significantly lower triglycerides compared to AS and AA females (*P* value < 0.001). Analysis of the triglycerides between AS individuals and AA controls revealed a significant difference between females in both groups (*P* value < 0.001) but no significant difference between males of both groups.

## 5. Discussion

To the best of our knowledge, this is the first study to elucidate the relation between plasma lipid profiles and hematological indices in Palestinian SCD patients. The Hb and RBC counts were significantly lower in SS and ST patients compared to SA individuals and AA controls (*P* value < 0.001). These findings agree with previous reports which indicate that SCD patients are generally anemic [3, 16].

Assessments of obesity using BMI was significantly (*P* value < 0.001) lower in the SCD patients than in those with no SCD (Table 1). The BMI results indicated that 95.5% of the studied participants were with normal weight (5^th^-84^th^) and only 4.5% were underweight (<5^th^) with the highest prevalence (14.5%) observed among patients with the Hb SS phenotype. Our results are inconsistent with the findings of Anto et al. and Osei-Yeboah et al. who reported 48.1% and 61.0% prevalence of malnutrition among children with SCD (underweight), respectively. [21, 22] The etiology of malnutrition in SCD has been associated with the chronic complications such as frequent crises, hepatomegaly, renal failure, anemia, and persistent infections. [23] It has been reported that patients with SCD had a nearly two-thirds decline in one or more growth parameters (weight, height, and BMI) and the incidence of growth retardation (defined by the presence of one or more of anthropometric parameters below the 5th percentile) could reach the 38% during the follow-up [24].

The knowledge of prognostic biomarkers in SCD may help to establish therapeutic interventions, management, late severe clinical complications, and follow-up in these patients. Several laboratory biomarkers that predict SCD clinical prognosis include fetal hemoglobin (HbF) concentration, leukocyte and reticulocyte counts, and lactate dehydrogenase (LDH) [25]. Here, in the present study, we hypothesized a possible link between severity of anemia and lipid profile in SCD patients. We measured CBC parameters and fasting lipid profile in our SS and ST patients as well as in AS carriers and age-sex-matched controls.

Hypocholesterolemia in SCD patients has been reported in many SCD cohort studies worldwide, yet the mechanism, pathophysiological manifestations, and association with mortality rate of these altered lipid levels have yet to be clarified [26–28]. Additionally, Song et al. have found that the cholesterol level has a U-shaped relation with mortality among Korean adult males and the probability of mortality is increased in patients with cholesterol levels below 135 mg/dL compared to those with cholesterol level between 135 and 200 mg/dL. [29]

In the present study, we examined serum levels of TC, HDL-C, LDL-C, and triglyceride in a cohort of SCD patients from Palestine and their relationships to severity of anemia and RBC indices. TC and LDL-C were significantly lower (*P* value < 0.001) in SS and ST patients compared to AS individuals and AA controls (Table 3). Decreased TC and LDL-C in SCD have been reported in many studies that examined lipids in SCD patients [16, 28, 30–35]. TC, in particular LDL-C, has a well-established role in atherosclerosis. The low levels of LDL-C in SCD are consistent with the low levels of total cholesterol and the virtual absence of atherosclerosis among SCD patients. SCD is characterized by a defect in RBC and plasma lipids associated with chronic oxidative stress, which may lead to atherosclerosis in these patients [30]. Lipids have been suggested to play a significant role in the pathophysiology of SCD, although the pathophysiological implications of these changes have not been completely resolved [25], and we think this issue needs to be reinvestigated in association with low levels of TC and LDL-C that have been reported in previous reports [28, 31–33] and confirmed in the present study. In our study, the mean TC in ST females and SS and ST males have TC levels below 135 mg/dL and thus should have an increased mortality rate based on the findings of Song et al. [29] Also, previous reports showed that adults and children with SCD tempt to have decreased levels of TC, HDL-C, and LDL-C and a significantly increased TG compared to age-sex-matched controls in normal population [28, 29, 34].

SCD patients may develop insulin resistance, which may be attributed to high reactive oxygen species (ROS) and increased ferritin level. ROS formation in SCD patients may be due to higher autooxidation of HbS generating hydrogen peroxide, which has deleterious consequences on lipid profile and serum glucose level [35]. Alsultan et al. reported significant higher fasting blood glucose (FBG) in SCD patients compared to the control. The authors claimed that an increase in the FBG level occurs as a consequence of insulin resistance developing among SCD patients. Sickle cell hemoglobin is associated with overproduction of reactive oxygen species (ROS). ROS may interfere with insulin signaling, impairing insulin uptake through a direct effect on insulin receptor function. ROS may reduce glucose uptake by inhibiting the translocation of GLUT4 (glucose transporter 4) to the plasma membrane leading to an increase in the serum blood glucose level. [35]

Besides chronic oxidative stress as a cause of reduced lipid profile in hemolytic anemia, another possible contributor could be an increase in cholesterol utilization and liver function abnormalities [16]. Marzouki et al. reported that increased cholesterol content per RBC is associated with decreased plasma or serum cholesterol in SCD [36]. In spite of t SCD hypocholesterolemia resulting from increased cholesterol utilization during the increased erythropoiesis of SCD, cholesterol is largely safeguarded through the enterohepatic circulation, at least in healthy people, and biosynthesis of new RBC membranes would likely use recycled cholesterol from the hemolyzed RBCs. In contrast, Westerman demonstrated that hypocholesterolemia was not due merely to increased RBC synthesis by showing that it is present in both hemolytic and nonhemolytic anemia. Thus, hypocholesterolemia appears to be a consequence of anemia itself rather than increased RBC biogenesis [37]. Another explanation is that hemolytic anemia in SCD leads to increased plasma volume and so dilution of its constituents including lipids and lipoproteins [30].

In our study, the HDL-C was significantly higher in SS and ST males and AS (males and females) compared to AA controls (*P* value < 0.001) which is consistent with other reports [16]. On the other hand, other reports had reported a reduction of HDL-C in the serum of children with SCD [38–40]. The potential reasons for inconsistencies among different studies include differences in age, gender, weight, diet, smoking, sample sizes, disease severity, and other underlying diseases and treatment regimens [41, 42].

The HDL-C plays important role in reducing the risk of hemolysis and improving endothelial dysfunction and contribute to a better clinical outcome. It was reported elsewhere that the downregulation of cholesterol biosynthesis occurs through a rate-limiting enzyme of *β*-hydroxymethyl-glutaryl-CoA reductase [31]. One of the mechanisms involving the reduction of cholesterol in SCD patients is lecithin cholesterol acyltransferase (LCAT). LCAT esterifies plasma cholesterol and lecithin molecules, the major phospholipid of HDL-C [43].

In our study, triglycerides in SS females were significantly lower than that in ST (*P* value < 0.05), AS (*P* value < 0.001), and AA controls (*P* value < 0.001). While in SS males, there was no significant difference in the triglyceride level when compared to AS individuals and AA controls. Previous reports by Ephraim et al. and Essohona et al. reported no significant difference in TG between SCD patients and controls [32, 44].

Triglycerides have not been as extensively studied as cholesterol in SCD, and previous studies have given diverse results. Increased triglycerides have been reported in several previous studies of SCD adults [45–47]; in addition, triglyceride levels were found to increase during crisis. Possible reasons for divergence among studies are similar to reasons for inconsistencies in HDL-C mentioned earlier. Furthermore, of all dietary lipids, triglycerides are the most dependent on fasting vs. nonfasting status of the patient at the time of blood collection, and in nonfasting patients, the amount and type of fat or fatty acids in the diet and time elapsed since the fats consumed can greatly influence the blood triglyceride levels.

## 6. Limitations of the Study

Our study has several important limitations. The majority of the patients included in this study are adolescents (late teens to early 20s), but children may have findings that may be important. The small size number of subjects in each group makes it difficult to draw concrete conclusion, and a larger number may be necessary for such purpose. The clinical data of the presence of atherosclerosis or coronary artery disease in the patients is lacking. Except for increased TG and decreased cholesterol levels, we also have no additional evidence of endothelial dysfunction and inflammatory state in our patients. Also, as large numbers of the controls were hospitalized patients, there was a possibility of bias which could have resulted in lipid values that were not representative.

The main outcome of our study was to find any relationship between dyslipidemia and hematological indices, in a cross-sectional way, to better understand the actual SCD disease severity in our region. Surely, a longitudinal study design and follow-up of patients will provide us more information. Still, it must be pointed that, in Palestine, SCD is a rare disease and, at the best of our knowledge, this is the first study on this topic.

## 7. Conclusion

In conclusion, we think that there is a reciprocal relationship between the erythrocyte and plasma parameters in SCD. In SCD, not only the erythrocyte but also the plasma is influenced in some way, particularly the plasma cholesterol which was examined in this study. In addition to hypocholesterolemia and LDL cholesterol, steady-state SCD patients were shown to have higher HDL and lower triglyceride levels in females. All these findings indicate that variations in lipid balance in SCD needs to be illustrated for their implication in the pathophysiology of the disease. We suggest that the relationship between oxidative hemolytic stress and hypocholesterolemia may be understood better by studying SCD patients in crisis during which the molecular pathology in HbS gives its most dramatic symptoms. Further prospective studies should examine the contribution of low plasma TC to the mortality rate in SCD patients.

## Figures and Tables

**Table 1:** General characteristics of study participants.

Characteristics	Total (*N* = 311)	Hb variant			Control (*N* = 160)	*P* value
		SS (*N* = 62)	ST (*N* = 54)	AS (*N* = 35)		
Age (years) (mean ± SD)	311	20.0 ± 11.10	17.5 ± 8.03^D,F∗^	21.8 ± 5.65^D∗^	21.7 ± 6.10^F∗^	<0.005
Age group (years), *n* (%)	311					
Children (≤13.9)	52 (16.7)	17 (27.4)	18 (33.3)	2 (5.7)	15 (9.4)	
Adults (≥14)	259 (83.3)	45 (72.6)	36 (66.7)	33 (94.3)	145 (90.6)	
Gender, *n* (%)	311					<0.157
Females	138 (44.4)	32 (51.6)	21 (38.9)	14 (40)	71 (44.4)	
Males	173 (55.6)	30 (48.4)	33 (61.1)	21 (60)	89 (55.6)	
BMI (kg/m^2^)	311	19.7 ± 1.53^B,C∗∗∗^	20.5 ± 1.77^D,F∗∗∗^	22.2 ± 1.62^B,D∗∗∗^	22.5 ± 1.57^C,F∗∗∗^	<0.001
BMI percentile						<0.001
Underweight (<5^th^)	14 (4.5)	9 (14.5)	3 (5.6)	0	2 (1.25)	
Normal weight (5^th^-84^th^)	297 (95.5)	53 (85.5)	51 (94.4)	34 (97.1)	158 (98.75)	
Overweight (≥85^th^)	1	0	0	1 (2.9)	0	

One-way ANOVA/compared means and Sig: ^A^^∗/∗∗^: SSXST; ^B∗/∗∗^: SSXAS; ^C^^∗/∗∗^: SSXCC; ^D^^∗/∗∗∗^: ASXST; ^E^^∗/∗∗^: ASXCC; ^F^^∗/∗∗^: STXCC. ^∗^: <0.05; ^∗∗^: <0.01; ^∗∗∗^: <0.001.

**Table 2:** Hematological parameters of SCD patients and controls. Data are expressed as mean ± SD. Mean comparison has been performed separately between the gender groups in patients and controls.

Group	*N*	Age (y)	Hb (g/dL)	RBC (×10^12^/L)	MCV (fL)	*P* value
Sickle cell anemia (SS)						
Females	32	21.2 ± 11.4	8.9 ± 0.6^a,b^	3.0 ± 0.3^a,b,c^	90.4 ± 3.2^a,b,c^	<0.001
Males	30	18.7 ± 10.9	9.2 ± 0.5^a,b^	3.4 ± 0.3^a,b,c^	91.9 ± 3.2^a,b,c^	
Subtotal	62					
Sickle *β*-thalassemia (ST)						
Females	21	19.2 ± 7.0	8.5 ± 1.3^a,b^	3.6 ± 0.6^a,b^	69.7 ± 7.3^a,b^	<0.001
Males	33	16.4 ± 8.5	8.9 ± 1.1^a,b^	3.9 ± 0.6^a,b^	71.1 ± 6.1^a,b^	
Subtotal	54					
Sickle cell trait (AS)						
Females	14	20.9 ± 3.6	12.3 ± 0.5	4.2 ± 0.2	84.2 ± 1.6	<0.05
Males	21	22.4 ± 6.7	14.3 ± 0.6^b^	4.8 ± 0.4^d^	86.6 ± 2.2	
Subtotal	35					
Controls (AA)						
Females	71	21.9 ± 7.0	12.5 ± 0.9	4.4 ± 0.5	81.9 ± 4.1	
Males	89	21.5 ± 5.4	15.1 ± 1.1	5.1 ± 0.5	86.5 ± 3.4	
Subtotal	160					
Total	311					

Statistically significant at *P* value < 0.001 compared to ^a^AS, ^b^AA, and ^c^ST or at *P* value < 0.05 compared to ^d^AA and ^e^ST.

**Table 3:** Lipid profile of SCD patients and controls. Data are expressed as mean ± SD. Mean comparison has been performed separately between the gender groups in patients and controls.

Group	*N*	Total cholesterol (mg/dL)	LDL cholesterol (mg/dL)	HDL cholesterol (mg/dL)	Triglycerides (mg/dL)	*P* value
Sickle cell anemia (SS)						
Females	32	136.4 ± 9.0^a,b^	72.9 ± 7.3^a,b,c^	42.4 ± 3.7^a,b,c^	105.3 ± 14.3^c^	<0.001
Males	30	109.8 ± 12.3^a,b^	56.5 ± 7.0^a,b^	36.7 ± 4.6^a,e^	83.1 ± 15.8^a,b,e^	
Subtotal	62					
Sickle *β*-thalassemia (ST)						
Females	21	131.6 ± 6.7^a,b^	58.6 ± 3.5^a,b^	48.6 ± 5.0^a,b^	122.1 ± 9.4^a,b^	<0.001
Males	33	111.3 ± 10.4^a,b^	54.7 ± 7.6^a,b^	38.6 ± 3.3^a^	89.6 ± 12.4^a,b^	
Subtotal	54					
Sickle cell trait (AS)						
Females	14	179.9 ± 6.0	99.2 ± 5.6^b^	59.1 ± 2.8^b^	103.9 ± 9.8	<0.001
Males	21	168.5 ± 11.6	89.5 ± 6.5^b^	55.6 ± 3.8^b^	121.8 ± 7.5^b^	
Subtotal	35					
Controls (AA)						
Females	71	180.6 ± 11.8	121.1 ± 9.4	38.7 ± 4.1	103.7 ± 13.5	
Males	89	171.1 ± 8.8	112.1 ± 6.6	37.6 ± 3.9	106.6 ± 11.8	
Subtotal	160					
Total	311					

Statistically significant at *P* value < 0.001 compared to ^a^AS, ^b^AA, and ^c^ST or at *P* value < 0.05 compared to ^d^AA and ^e^ST.

## Data Availability

The data used to support the findings of this study are available from the corresponding author upon request.
